# Efficacy and Safety of Escitalopram Oral Drops to Treat Major Depressive Disorder and Generalized Anxiety Disorder in Adolescent, Adult and Geriatric Patients: A Prospective Multicenter Observational Study in Pakistan

**DOI:** 10.7759/cureus.6792

**Published:** 2020-01-27

**Authors:** Muhammad Iqbal Afridi, Imtiaz Ahmad Dogar, Asad T Nizami, Rubina Aslam, Ali Burhan Mustafa, Sharib Syed Muhammad, Neeta Maheshwary

**Affiliations:** 1 Psychiatry and Behavioral Sciences, Jinnah Postgraduate Medical Center, Karachi, PAK; 2 Psychiatry and Behavioral Sciences, Punjab Medical College, DHQ Hospital, Faisalabad, PAK; 3 Psychiatry, Rawalpindi Medical University, Benazir Bhutto Hospital, Rawalpindi, PAK; 4 Psychiatry, Jinnah Hospital, Lahore, PAK; 5 Psychiatry and Behavioral Sciences, Sheikh Zayed Medical College and Hospital, Rahim Yar Khan, PAK; 6 Clinical Research, Hilton Pharma (Pvt.) Ltd., Karachi, PAK

**Keywords:** escitalopram oral drops, generalized anxiety, ham-a, major depressive disorder, madrs

## Abstract

Background

Escitalopram is widely used for the management of the major depressive disorder and generalized anxiety disorder, but there is no to very limited data available regarding efficacy and safety in Pakistani patients. This study was conducted to evaluate the efficacy and safety of escitalopram oral drops to manage the major depressive disorder and generalized anxiety disorder in a local cluster within Pakistan.

Methods

This prospective multicenter observational study was conducted in the department of psychiatry from August 2018 - August 2019. Eighty-five patients meeting the selection criteria were included in the study. Adolescent, adult, and geriatric patients of either gender with generalized anxiety disorder having Hamilton Anxiety Rating Scale (HAM-A) rate ≥ 10 and major depressive disorder having Montgomery-Asberg Depression Rating Scale (MADRS) rate ≥ 7 or patients with co-morbid generalized anxiety disorder (GAD), major depressive disorder (MDD) were selected for the study. We are reporting patients’ improvement from baseline, response rate, and remission rate. Data analysis is performed by using SPSS version 21 (IBM Inc, Armonk, USA).

Results

Among enrolled patients, 42 were adolescents, 22 were adults, and 21 were geriatric. The mean age of an adolescent, adult, and geriatric patients was 14.92 ± 2.04, 44.54 ± 12.08, and 64.61 ± 3.16 years, respectively. Among enrolled patients, the mean change in a total score of HAM-A for anxiety and MADRS for depression were -10.04 ± 4.32 and -17.67 ± 14.42, respectively. At the end of the study, the remission rate and response rate for depression were 82 % and 75%, respectively. Similarly, the remission rate and the response for anxiety were 76% and 81%, respectively. Mean HAM-A and MADRS scores were significantly improved for adolescent, adult, and geriatric patients. Adverse events were reported in eight (9.41%) patients with six having gastrointestinal (GI) disturbance and two having to worsen anxiety. All reported adverse events were of mild severity.

Conclusion

Escitalopram oral drops are found effective and tolerable in reducing both anxiety and depression over the duration of study in all age groups, including adolescents, adults, and geriatrics.

## Introduction

Among all the mood and anxiety disorders, major depressive disorder (MDD) and generalized anxiety disorder (GAD) constitute the highest rate of co-morbidity [1]. The ranges of co-morbidity in studies have been reported as low as 40% to as high as 98% [2]. In epidemiological studies such as the National Comorbidity Survey (NCS), it is mentioned that in 67% of patients with a lifetime GAD, retrospectively have reported MDD while around 20% of patients with MDD retrospectively have been observed with GAD [3]. A common symptom i.e. general distress is seen in the majority of patients with mood and anxiety disorders. Nonetheless, every disorder has its own characteristic feature. For instance, excessive worrying in several situations is a characteristic of GAD while anhedonia (inability to feel pleasure in normally pleasurable activities) is a feature of MDD [4].

Excessive distress in normal situations is a feature of GAD. It is a common and disabling disorder. In GAD patients, their likelihood is over-estimated and also their negative consequences are thought to be catastrophic [5]. The feeling of distress can very rapidly become a routine in daily life and affect those who suffer from GAD, affecting their social activities as well. Health, familial relationships, financial or occupational situations can all be affected. Such worries normally lead to defensive and avoidant behavior [6].

MDD is also a commonly observed disabling condition having a high frequency of failure to recover from the disorder [7]. This leads to increased rates of morbidity and mortality. In accordance with the report of the World Health Organization (WHO), the worldwide incidence of MDD was estimated to be around 4.4% in 2015 [8]. Anti-depressants are the mainstay of treatment in MDD and in other mood and anxiety disorders. Compliance can be achieved by administrating bi-weekly doses [9]. Despite the availability of a wide variety of anti-depressant drugs, most clinical researches have reported that around one-third of patients having MDD minimally respond or fail to respond to the first-line of anti-depressant treatment. This can occur even when the dose and duration of drug treatment are adequate [10]. A dire need exists for the urgent development of novel and improved drugs or drug combinations for treating depression.

Depression is not only a serious ailment resulting in substantial disability but is also associated with high morbidity and an increased risk of suicide [11]. Researches have suggested that anti-depressants which act on both nor-adrenergic and serotonergic receptor systems have been found to be highly effective in comparison to selective serotonin reuptake inhibitors (SSRIs) [12]. SSRIs specifically bind to the serotonergic receptor system. Nevertheless, escitalopram is an allosteric SSRI that binds to both primary site of serotonin transporter system as well as to an allosteric site which greatly augments the efficacy of serotonin reuptake inhibition [13].

Almost all guidelines regarding depression treatment have recommended that treatment should be continued for at least six to nine months following remission. In majority of patients, a six-month treatment plan is considered as endorsed by the National Institute for Clinical Excellence (NICE) in United Kingdom [14].

General anxiety disorder is scored using the Hamilton Anxiety Rating Scale (HAM-A) which is a self-employed clinician based questionnaire consisting of 14 symptoms that cater both somatic as well as psychological features [15]. Likewise, the scoring of Major Depressive Disorder is done using the Montgomery-Asberg Depression Rating Scale (MADRS) [16].

The objective of this study was to evaluate the safety and efficacy of escitalopram oral drops for treating the major depressive disorder and generalized anxiety disorder in adolescent, adult, and geriatric patients. The study aided in gathering real-life setting clinical data among the local population. 

## Materials and methods

This prospective, multicenter, observational study was conducted in the department of psychiatry from August 2018 to August 2019. Ethical approval was obtained from the Institutional Review Board of Jinnah Postgraduate Medical Center. The Study follows Strengthening the Reporting of Observational Studies in Epidemiology (STROBE) guidelines to report the results of the study.

Settings and participants

Eighty-five patients visiting the department of psychiatry of Jinnah Postgraduate Medical Center (Karachi), DHQ Hospital (Faisalabad), Benazir Bhutto Hospital (Rawalpindi), Jinnah Hospital (Lahore), and Sheikh Zayed Medical College Hospital (Rahim Yar Khan) were included in the study after getting written consent and meeting selection criteria. Patients were included using a non-probability convenient sampling technique.

Adolescent, adult and geriatric patients of either gender with GAD having HAM-A scale rating ≥ 10 and MDD having MADRS ≥ 7 or patients with co-morbid GAD, MDD having HAM-A and MADRS of ≥10 and ≥7 respectively were selected for the study. Patients using monoamine oxidase inhibitors with 14 days of escitalopram treatment, with linezolid or IV methylene blue treatment, pregnant mothers and patients having a seizure disorder, bipolar mania, depression or severe renal impairment and patients hypersensitive to escitalopram or any of its ingredients were excluded from the study.

Study process

Patients were followed up for a period of 16 weeks during which data was gathered through medical record and any adverse event (AE) or severe adverse event during the course of the study were recorded on a case report form (CRF) for patients who were prescribed with escitalopram oral drops (Citanew® oral drops) 10 mg/day dissolved in 250 mL glass of water for 16 week period.

HAM-A classification was used for GAD which consisted of 14 items and each numbered as zero for no symptom, one as mild, two as mild-moderate, three as a moderate and four as a severe symptom. A total score of <17 was recorded as a patient having mild severity, between 18-24 as mild to moderate severity and score between 25-30 as moderate to severe GAD. Similarly, MADRS classification was used for MDD that consisted of 10 items with scores of zero, two, four and six according to symptom severity. A total MADRS score in-between 9-17 was regarded as mild, in-between 18-34 as moderate and >35 as severe MDD. For both HAM-A and MADRS, scoring was done at baseline, week 2, week 8 and week 16.

Statistics

Data was analyzed using SPSS version 21 (IMB Inc, Armonk, USA). For quantitative data, mean and standard deviation were reported and for qualitative data frequencies and percentages were presented. A line graph was used to assess the difference. A paired t-test was used to assess the significance and a p-value of ≤0.05 was set as a significant level.

## Results

Eighty-five patients were enrolled in the study. Forty-two were adolescents, 22 were adults, and 21 were geriatrics. The mean age of an adolescent, adult, and geriatric patients was 14.92 ± 2.04, 44.54 ± 12.08, and 64.61 ± 3.16 years, respectively. Among enrolled patients, 45 (52.3%) were females. The mean body mass index (BMI) of all study patients was 22.41±5.87 kg/m2. Patients were diagnosed as having major depressive disorder alone (29.7%), generalized anxiety disorder alone (15.3%), or co-morbid MDD and GAD (57.14%). Baseline characteristics are listed in Table 1.

**Table 1:** Baseline characteristics: adolescent, adult and geriatric patients Mean ± SD for continuous variables and n (%) for descriptive variables. MDD - major depressive disorder; MADRS - Montgomery-Asberg Depression Rating Score; GAD - generalized anxiety disorder; HAM-A - Hamilton Anxiety Rating

		Adolescent (n=42)	Adult (n=22)	Geriatric (n=21)
Variables		Mean ± SD / n (%)	Mean ± SD / n (%)	Mean ± SD /n (%)
Age (years)		14.92 ± 2.04	44.54 ± 12.08	64.61 ± 3.16
Gender	Male	22 (52.4%)	10 (45.5%)	9 (42.9%)
	Female	20 (47.6%)	12 (54.5%)	12 (57.1%)
Body mass index (kg/m^2^)		19.41 ± 4.63	30.85 ± 3.04	26.53 ± 4.67
Diabetes		Nil	5 (22.7%)	7 (50.0%)
Smoking		2 (6.3%)	4 (18.2%)	1 (7.1%)
Hematologic disease		Nil	Nil	Nil
Steroid intake		6 (19.4%)	1 (4.8%)	1 (7.1%)
Hypertension		Nil	4 (21.1%)	8(61.5%)
MDD Only		14 (36.8%)	4 (25.0%)	7 (38.9%)
GAD Only		6 (15.8%)	Nil	5 (27.8%)
Mixed MDD and GAD disorder		21 (51.2%)	18 (81.8%)	9 (45.0%)
Depression at baseline (MADRS)	Mild	3 (8.1%)	1 (4.5%)	2 (12.5%)
	Mild-moderate	28 (75.7%)	9 (40.9%)	9 (56.2%)
	Severe	6 (16.2%)	12 (54.6%)	5 (31.3%)
Anxiety at baseline (HAM-A)	Mild	19 (45.2%)	5 (22.7%)	7 (33.3%)
	Mild-moderate	14 (33.3%)	3 (13.6%)	5 (23.8%)
	Moderate-severe	9 (21.4%)	14 (63.7%)	9 (42.9%)

Prior to initiation of treatment with escitalopram oral drops, 17.6% of patients had been treated with at least one of the following: SSRIs (n = 14), benzodiazepines (n=12) tetracyclic antidepressants (n=2), psychotherapy (n=1).

Among enrolled patients, the mean change in the total score of HAM-A for anxiety and MADRS for depression were -10.04 ± 4.32 and -17.67 ± 14.42, respectively. At the end of the study, the remission rate (MADRS ≤ 12) and response rate (≥ 50 % decrease in MADRS score) for depression was 82% and 75%, respectively. Similarly, the remission rate (HAM-A ≤ 10) and response rate (≥ 50 % decrease in HAM-A score) for anxiety was 76 % and 81%, respectively (see Table 2).

**Table 2:** Diﬀerence in total scores remission and response rates for MADRS and HAM-A in all patients Negative numbers indicate an improvement. Response rate: ≥ 50 % improvement from baseline. Remission rate: MADRS ≤ 12, HAM-A ≤ 10. HAM-A - Hamilton Anxiety Scale; MADRS - Montgomery-Asberg Depression Rating Scale

Variables	MADRS	HAM-A
Mean change in total score	- 17.67 ± 14.42	- 10.04 ± 4.32
Response rate	75%	81%
Remission rate	82%	76%

**Figure 1:**
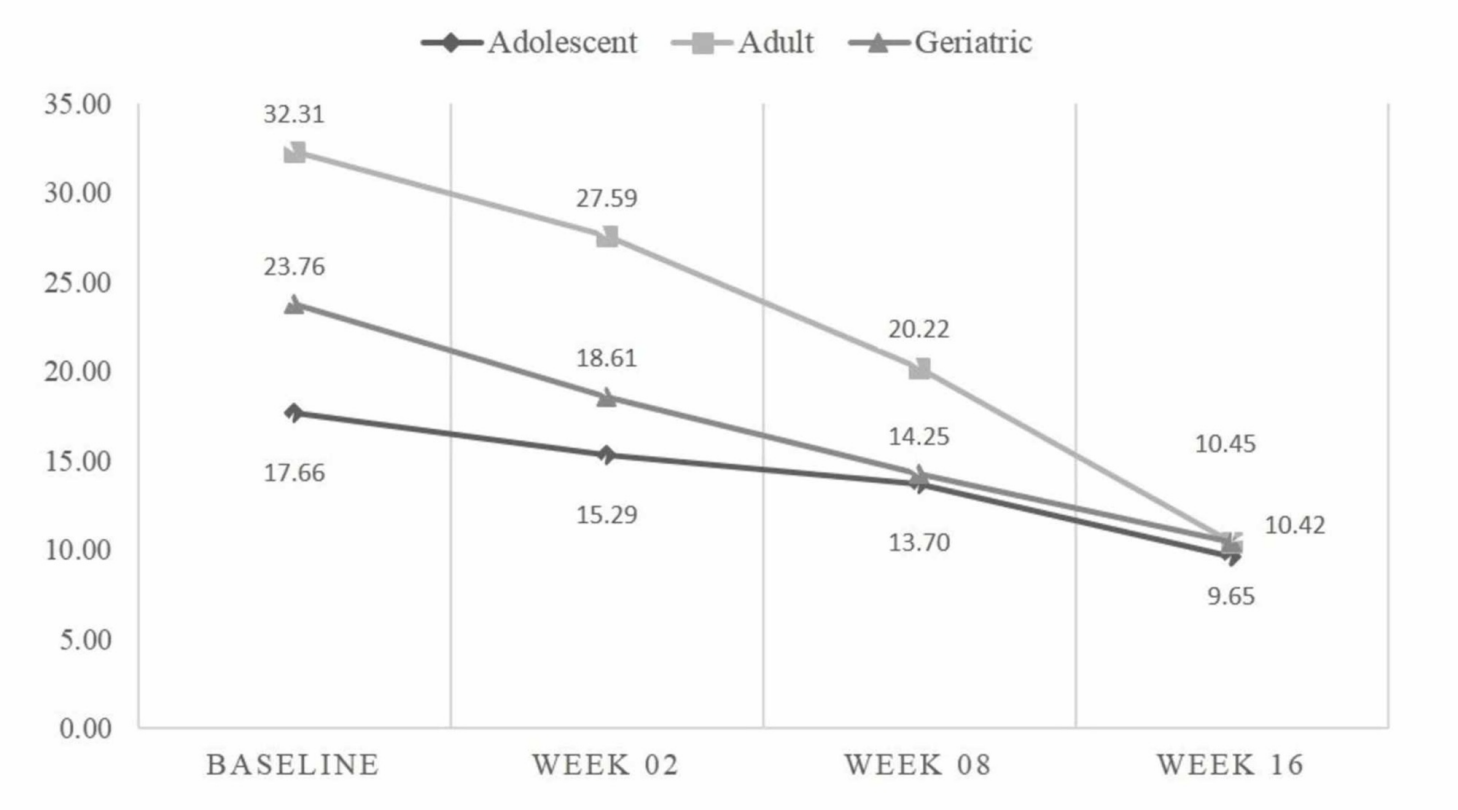
Comparison of mean HAM-A scores at different study periods HAM-A - Hamilton Anxiety Scale

**Figure 2:**
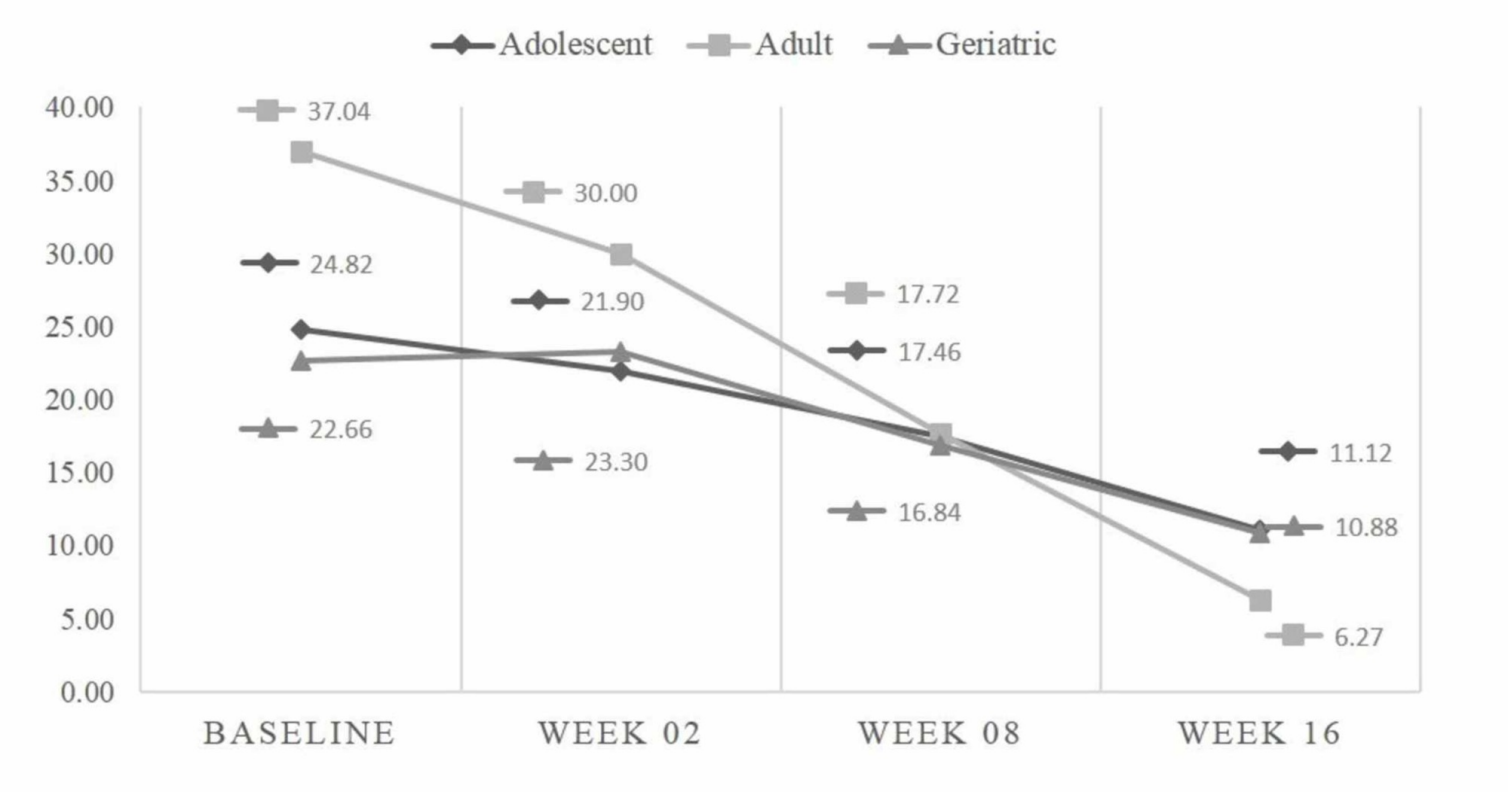
Comparison of mean MADRS at different study periods MADRS - Montgomery-Asberg Depression Rating Scale

Among 42 adolescents, the mean baseline HAM-A score was 18.10 ± 7.8, while at 16^th^ week, it was reduced to 9.66 ± 4.18 (p<0.001). The mean MADRS score at baseline was 24.83 ± 9.38, while at the 16^th^ week it was 11.12 ± 6.96 (p<0.001). Among 22 adults, the mean baseline HAM-A score was 32.32 ± 15.01, while at the 16^th^ week it was 10.45 ± 5.28 (p-<0.001). The mean MADRS score at baseline was 37.05 ± 15.45, while at the 16^th^ week it was 6.27 ± 4.71 (p<0.001). Among 21 geriatric patients, the mean baseline HAM-A score was 23.74 ± 10.46, while at the 16^th^ week it was 10.42 ± 3.46 (p<0.001). The mean MADRS score at baseline was 22.56 ± 11.63, while at the 16^th^ week it was 10.89 ± 5.14 (p<0.001) (see Table 3). Mean HAM-A and MADRS scores at baseline, week 2, week 8, and week 16 for adolescent, adult, and geriatric patients are depicted in Figures 1 and 2.

**Table 3:** Comparison of mean anxiety and depression scores baseline to week 16: adolescent, adult and geriatric patients A paired sample t-test was used to check the significance at α = 0.05. CI - confidence interval; SD - standard deviation; HAM-A - Hamilton Anxiety Scale; MADRS - Montgomery-Asberg Depression Rating Scale

Variables		Mean ± SD	95% CI	p-value
Adolescents (n=42)				
HAM-A	Baseline score	18.10 ± 7.80	15.63 - 20.56	<0.001
	Week 16 score	9.66 ± 4.18	8.33 - 10.98	
MADRS	Baseline score	24.83 ± 9.38	21.86 - 27.79	<0.001
	Week 16 score	11.12 ± 6.96	8.92 - 13.32	
Adults (n=22)				
HAM-A	Baseline score	32.32 ± 15.01	25.66 - 38.97	<0.001
	Week 16 score	10.45 ± 5.28	8.11 - 12.79	
MADRS	Baseline score	37.05 ± 15.45	30.19 - 43.89	<0.001
	Week 16 score	6.27 ± 4.71	4.18 - 8.36	
Geriatrics (n=21)				
HAM-A	Baseline score	23.74 ± 10.46	18.69 - 28.77	<0.001
	Week 16 score	10.42 ± 3.46	8.74 - 12.09	
MADRS	Baseline score	22.56 ± 11.63	16.76 - 28.34	<0.001
	Week 16 score	10.89 ± 5.14	8.33 - 13.44	

Adverse events were reported in 8 (9.41%) patients with six having gastrointestinal (GI) disturbance and two having worsening of anxiety. All reported adverse events were of mild severity. No serious adverse event was reported during the course of the study. 

## Discussion

The therapeutic eﬃcacy of escitalopram in patients with depression or diﬀerent anxiety disorders was demonstrated in several clinical studies [17, 18]. This prospective observational study confirmed the use of escitalopram oral drops as effective and tolerable in adolescent, adult and geriatric patients with MDD and GAD or co-morbid MDD and GAD. In our study findings, most of the patients having anxiety and/or depression were of moderate to severe severity.

The response rate and remission rate for HAM-A in anxiety and MADRS in depression in our study results are comparable to previously published rates [19, 20]. The magnitude of the average drop in the total HAM-A and MADRS score after 16 weeks of treatment with oral escitalopram drops, among adolescents, adults as well as in geriatric patients has shown a significant effect in managing or treating both major depression and generalized anxiety. Age-specific (adolescent, adults and geriatrics) improvement in HAM-A and MADRS is also evident from G. Laux et al. study [21].

Similarly, one of the studies reported a significant drop in mean HAM-A and MADRS score which used escitalopram for treatment [17]. Few studies from other countries have also reported that escitalopram has an effective treatment potential in decreasing the effects of anxiety [18]. A meta-analysis that compared the safety and effectiveness of 12 most commonly used anti-depressants found that escitalopram was one of those anti-depressants which showed the best treatment effects and had a high rate of adherence to treatment [22]. Results from a Chinese study in which patients with moderate to severe episodes of major depression seeking treatment were given escitalopram and showed positive results [23].

Escitalopram and other SSRI have the highest advantage of having no dependency, being safe in the case of overdoses and easily tolerable side effects, however, few disadvantages include a latency period of two to six weeks and treatment leads to agitation and nervousness initially [24]. The findings of the above studies are consistent with our results.

In another study by Maneeton et al., escitalopram was reported to significantly decrease the HAM-A and MADRS scores of anxiety and depression following treatment. Compared with duloxetine, escitalopram was reported to have superior tolerability, fewer dropout rate and better compliance of patients [25].

Very rare adverse events were reported in our study, which is also reported in published studies that for escitalopram and other SSRI’s of its class were reported to be better tolerated with fewer adverse effects, with most adverse effects occurred at the start of treatment and subsided gradually [3].

The mixed approach of our study has ascertained that we have sampled an extension range of patients. However, the study might not be immune to selection and observer bias. Considering the observations of our study and to what extent the treatment will be comparable with combination therapies would be enlightening to discover more facts about the treatment of depression and anxiety disorders.

## Conclusions

Based on the study results it can be concluded that escitalopram oral drops significantly reduced both anxiety and depression over the duration of study in adolescent, adult, and geriatric patients. Escitalopram oral drops can be recommended as an effective treatment option whenever management of anxiety and/or depression among adolescent, adult or geriatric patients is considered specially whenever there is compliance concern with oral tablets.
